# Response: Commentary: Human gut, breast, and oral microbiome in breast cancer: A systematic review and meta-analysis

**DOI:** 10.3389/fonc.2023.1279862

**Published:** 2023-10-30

**Authors:** May Soe Thu, Krit Pongpirul

**Affiliations:** ^1^ Joint Chulalongkorn University - University of Liverpool Ph.D. Programme in Biomedical Sciences and Biotechnology, Faculty of Medicine, Chulalongkorn University, Bangkok, Thailand; ^2^ Center of Excellence in Immunology and Immune-Mediated Diseases, Department of Microbiology, Faculty of Medicine, Chulalongkorn University, Bangkok, Thailand; ^3^ Department of Infection Biology and Microbiomes, Faculty of Health and Life Science, University of Liverpool, Liverpool, United Kingdom; ^4^ Center of Excellence in Preventive & Integrative Medicine, Department of Preventive and Social Medicine, Faculty of Medicine, Chulalongkorn University, Bangkok, Thailand; ^5^ School of Global Health, Faculty of Medicine, Chulalongkorn University, Bangkok, Thailand; ^6^ Department of International Health, Johns Hopkins Bloomberg School of Public Health, Baltimore, MD, United States; ^7^ Clinical Research Center, Bumrungrad International Hospital, Bangkok, Thailand

**Keywords:** microbiome, breast cancer, cancer staging, standard deviation, standard error

## Introduction

We greatly appreciate the insightful commentary that accompanied our recent systematic review, titled “Human gut, breast, and oral microbiome in breast cancer: A systematic review and meta-analysis” (1). The thoughtful remarks not only highlight the importance of our work but also contribute significantly to the broader dialogue surrounding microbiome-focused systematic reviews. We are grateful for the meticulous examination conducted by esteemed colleagues, as it undoubtedly enriches the credibility of our findings.

## Commentary and discussion

Regarding the classification of breast cancer subtypes, we acknowledge the clarification provided. The AJCC Cancer Staging approach by Amin et al. (2017) utilizes a TNM-based system for assessing disease extent on a population level (2), while the molecular hallmarks-based classification for breast cancer by Loibl et al., 2021 is indeed the accurate concept (3). We appreciate your reference to the work by Orrantia-Borunda et al. (2022) that sheds light on the utilization of HER2 markers in classifying luminal A and B subtypes (4).

The need for correction in Table 5 is well noted, and it needs to be revised as “gynecological cancer”. With regard to Tables 3-5, we recognize your point about presenting microbiota detection methods in greater detail, specifying various sequencing techniques. We chose to highlight sequencing platforms to maintain clarity for general readers, given our primary focus on the microbiota findings in breast cancer.

We concur fully with the observation regarding the use of different measures of dispersion in the cited studies. The discrepancies between standard error in the study by Goedert in 2015 (5) and standard deviation in the study by Byrd in 2021 (6) were unintended oversights during our analysis. It is evident that data transposition errors of this nature are not uncommon in the realm of meta-analysis (7). The review conducted by Daniel et al. not only emphasized the pervasiveness of such concerns but also underscored the imperative of upholding meticulous statistical accuracy. Their findings shed light on the prevalence of these issues, revealing that within the domain of strength and conditioning research, nearly 85% of the 20 most extensively referenced meta-analyses were plagued by statistical inaccuracies. Of note, almost half of these studies (45%) contained at least one instance where effect sizes had been erroneously calculated using standard error instead of standard deviation (8).

This commentary underscores our commitment to scientific rigor and precision. It has also highlighted several key takeaways: the importance of accurately conveying information and referencing sources in the introduction, the sensitivity of data input in meta-analysis due to unit differences; and the potential benefits of providing more sequencing method details for a comprehensive view of microbiome diversity.

In conclusion, we primarily followed the PRISMA reporting guidelines (9) for our review. However, we recognize that there were aspects, particularly related to data items and synthesis methods, where data unit checks were lacking. We acknowledge the potential for improvement in future PRISMA reports to enhance accuracy and conciseness. Through this discourse, our aim is to fortify the foundation of microbiome studies, facilitating a deeper understanding of the intricate connections that underlie breast cancer’s development and progression. Thank you once again for the meticulous analysis and valuable contribution to advancing the quality of scientific research in this field.

## Author contributions

MT: Conceptualization, Data curation, Formal Analysis, Investigation, Visualization, Writing – original draft, Writing – review & editing. KP: Conceptualization, Methodology, Project administration, Resources, Supervision, Writing – original draft, Writing – review & editing.
